# Soy and fish as features of the Japanese diet and cardiovascular disease risks

**DOI:** 10.1371/journal.pone.0176039

**Published:** 2017-04-21

**Authors:** Yukio Yamori, Miki Sagara, Yoshimi Arai, Hitomi Kobayashi, Kazumi Kishimoto, Ikuko Matsuno, Hideki Mori, Mari Mori

**Affiliations:** 1 Mukogawa Women’s University Institute for World Health Development, Hyogo Japan; 2 Laboratory of Preventive Nutritional Medicine, Research Institute for Production and Development, Kyoto, Japan; 3 Hyogo Prefecture Health Promotion Association, Kobe, Hyogo, Japan; The University of Tokyo, JAPAN

## Abstract

In the World Health Organization (WHO)-coordinated Cardiovascular Disease and Alimentary Comparison Study, isoflavones (I; biomarker for dietary soy) and taurine (T; biomarker for dietary fish) in 24-hour—urine (24U) were inversely related to coronary heart disease (CHD) mortality. High levels of these biomarkers are found in Japanese people, whose CHD mortality is lowest among developed countries. We analyzed the association of these biomarkers with cardiovascular disease risk in the Japanese to know their health effects within one ethnic population. First, to compare the Japanese intake of I and T with international intakes, the ratios of 24UI and 24UT to creatinine from the WHO Study were divided into quintiles for analysis. The ratio for the Japanese was the highest in the highest quintiles for both I and T, reaching 88.1%, far higher than the average ratio for the Japanese (26.3%) in the total study population. Second, 553 inhabitants of Hyogo Prefecture, Japan, aged 30 to 79 years underwent 24-U collection and blood analyses. The 24UT and 24UI were divided into tertiles and adjusted for age and sex. The highest T tertile, compared with the lowest tertile, showed significantly higher levels of high-density lipoprotein-cholesterol (HDL-C), total cholesterol, 24U sodium (Na) and potassium (K). The highest I tertile showed significantly higher folate, 24UNa and 24UK compared with the lowest tertile. The highest tertile of both T and I showed significantly higher HDL-C, folate, and 24UNa and 24UK compared with the lowest tertile. Thus, greater consumption of fish and soy were significantly associated with higher HDL-C and folate levels, possibly a contributor to Japan having the lowest CHD mortality and longest life expectancy among developed countries. As these intakes were also associated with a high intake of salt, a low-salt intake of fish and soy should be recommended for healthy life expectancy.

## Introduction

Healthy aging is a goal of rapidly aging populations. Japanese people, particularly women, have enjoyed the world’s longest average life expectancy since 1985 [1,2].

Since nutrition is closely related to the risk of chronic disease, including cardiovascular diseases, it affects healthy aging [3–5]. The Japanese diet which was recently featured on the UNESCO's Representative List of the Intangible Cultural Heritage of Humanity is regarded as being good for healthy aging, together with the Mediterranean diet, because of their association with lower mortality rates from coronary heart disease (CHD) in Japanese and Mediterranean populations [6,7]. Japanese and Mediterranean diets have similar features of customarily eating seafood, vegetables, fruits, and nuts or beans [2–4]. Nuts are not commonly eaten in Japan; however, soybeans and soy products are popular. These common foods in both diets contain magnesium. Magnesium in 24-hour urine (24U) was proved to be inversely related to cardiovascular risk factors such as obesity, hypertension, and hypercholesterolemia in the WHO-coordinated Cardiovascular Disease and Alimentary Comparison (CARDIAC) Study [8] and supported the nutritional merit of both diets for longevity.

The CARDIAC Study, a worldwide health examination started in 1985, revealed that lower CHD mortality rates were related to longer average life-expectancies and that CHD mortality rates of the study populations were significantly inversely related to the average excretion levels of taurine (T) and isoflavones (I) (biomarkers of fish and soy intake, respectively) in 24U in these populations [8–11]. Since these data indicate that a higher intake of fish and soy may be related to the longer life expectancy of the Japanese due to their effects on the risk reduction of CHD, we first evaluated the consumption of these nutritional components by Japanese in comparison to international consumption levels. Second, we investigated the association of fish and soy intakes with CHD risk in the Japanese using 24U analysis of T and I.

## Materials and methods

From the CARDIAC study, 851 24U samples from male and female study participants aged 48–56 years in 50 populations [8] were analyzed for both T and I, biomarkers of fish and soy intakes, respectively. The creatinine ratios of T and I were divided into quintiles, T1–5 and I1–5. The ratio of Japanese to the total in each one of 25 groups (5 x 5 quintiles) was calculated. Chi-square tests for trend were performed among 5 groups, T1I1 (the lowest T and lowest I), T2I2, T3I3, T4I4 and T5I5 (the highest T and highest I).

Male and female inhabitants, aged 30–79 years were invited to health examinations held by 10 local health offices distributed throughout city, coast, and mountain areas of Hyogo Prefecture in 2011 and 2012 [12]. Hyogo Prefecture is located in the middle of Japan, with a population of about 5.6 million, i.e., one-twentieth of the total Japanese population. Written informed consent was obtained from all participants prior to inclusion. A total of 553 participants (including 322 female participants) underwent anthropometrical and blood pressure measurements using an automated system (Omron HEM 907), fasting blood sampling, and 24U collection by using aliquot cups [13], and completed questionnaires on dietary habits and medical history. Blood samples were collected for lipid profile (total cholesterol [TC], high density lipoprotein-cholesterol [HDL-C], and low density lipoprotein-cholesterol [LDL-C], triglyceride, and folate levels; 24U samples were analyzed for sodium (Na) to calculate salt intake as well as potassium (K), T, and I to estimate the intake of vegetable, seafood, and soy, respectively. Participants were divided into tertiles, according to the creatinine ratios of T (T1–3) or of I (I1–3). General linear models were used to estimate adjusted mean values of body mass index (BMI), blood pressure, and serum and urinary biomarkers across T1–3, I1–3, or its combination (T1I1–T3I3), and multiple linear regression models were used to estimate the linear trend in each analysis.

All analyses were done using IBM SPSS software (version 19, IBM Corp., Armonk, NY, USA). Two-tailed P < 0.05 was considered statistically significant.

The study protocol using human samples was approved by the ethical committee, Mukogawa Women’s University (No. 13–04). All clinical investigations have been conducted according to the principles expressed in the Declaration of Helsinki.

## Results

### Japan’s high consumption of fish and soy

**Fig 1:**
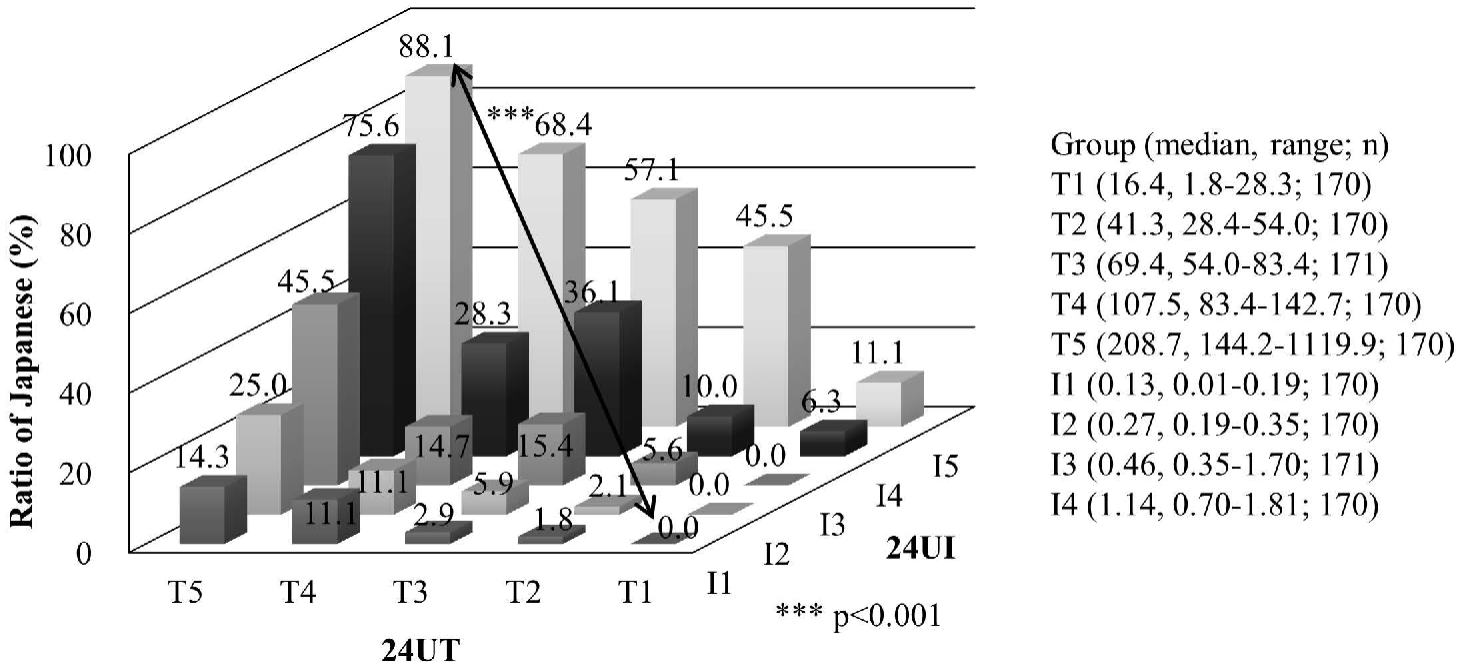
The ratio of Japanese in 25 groups divided into the quintiles of 24U T and I, the biomarkers of fish and soy intakes.

The creatinine ratios of T and I were divided into quintiles, T1–5 and I1–5, and arranged from low to high values. The ratios of Japanese people in each group were compared in Fig 1.

From the groups T1I1, T2I2, T3I3, T4I4, and T5I5, the ratios of Japanese were 0, 2.1, 15.4, 28.3, and 88.1%, respectively. The top T5I5 group included nearly 90% Japanese, which is far higher than the average ratio of 26.3% Japanese in the total CARDIAC study population, indicating this population is customarily taking higher amounts of fish and soy.

### Fish and soy intakes and cardiovascular risks in Japanese

#### Fish intake and cardiovascular risks

Male and female participants aged 30 to 79 years, (303 for whom all the data were available) were divided into tertiles T1–T3. Their data, the mean ± standard error, are shown in Table 1.

**Table 1:** Association of tertiles of biomarker of fish intake (24UT) with cardiovascular risks, fasting blood, and 24U in Japanese, Hyogo inhabitants aged 30 to 79 Years.

	Tertile of 24UT/Creatinine ratio, μmol/mmol			P for linear trend^1^
	1 (low)	2	3 (high)	
Median	62.9	123.0	233.6	
Range	18.8–93.5	96.2–159.2	159.3–1146.7	
Body mass Index				
n	101	101	101	
Crude	22.5 ± 0.3^2^	22.9 ± 0.3	21.9 ± 0.3	NS
Age and gender adjusted	22.6 ± 0.3	22.8 ± 0.3	22.0 ± 0.3	NS
Systolic blood pressure (mmHg)				
n	101	101	101	
Crude	120.4 ± 1.8	125.3 ± 1.6	122.1 ± 1.6	NS
Age and gender adjusted	122.0 ± 1.5	124.0 ± 1.5	121.8 ± 1.5	NS
Diastolic blood pressure (mmHg)				
n	101	101	101	
Crude	72.1 ± 1.2	74.9 ± 1.2	72.9 ± 1.1	NS
Age and gender adjusted	72.5 ± 1.1	74.2 ± 1.1	73.1 ± 1.1	NS
Serum total cholesterol (mg/dL)				
n	101	101	101	
Crude	198.3 ± 3.3	208.4 ± 3.4	213.5 ± 3.7	p<0.01
Age and gender adjusted	201.2 ± 3.3	207.2 ± 3.3	211.8 ± 3.3	p<0.05
Low-density lipoprotein cholesterol (mg/dL)				
n	101	101	101	
Crude	117.8 ± 2.9	126.8 ± 3.2	125.0 ± 3.4	NS
Age and gender adjusted	120.6 ± 3.0	125.4 ± 3.0	123.5 ± 3.0	NS
High-density lipoprotein cholesterol (mg/dL)				
n	101	101	101	
Crude	61.1 ± 1.4	60.8 ± 1.3	67.3 ± 1.6	p<0.01
Age and gender adjusted	60.6 ± 1.4	61.5 ± 1.4	67.0 ± 1.4	p<0.001
Triglyceride (mg/dL)				
n	96	97	99	
Crude	107.8 ± 8.2	114.8 ± 7.8	95.4 ± 5.2	NS
Age and gender adjusted	110.8 ± 7.1	111.3 ± 7.0	95.8 ± 6.9	NS
Folate (ng/mL)				
n	101	101	101	
Crude	6.5 ± 0.3	6.6 ± 0.3	7.4 ± 0.4	NS
Age and gender adjusted	6.7 ± 0.3	6.6 ± 0.3	7.2 ± 0.3	NS
24U Salt (g/day)				
n	101	101	101	
Crude	10.2 ± 0.5	11.8 ± 0.5	11.6 ± 0.4	p<0.05
Age and gender adjusted	10.1 ± 0.4	11.6 ± 0.4	11.8 ± 0.4	p<0.01
24U potassium (g/day)				
n	101	101	101	
Crude	1.8 ± 0.1	2.0 ± 0.1	2.1 ± 0.1	p<0.01
Age and gender adjusted	1.9 ± 0.1	2.0 ± 0.1	2.1 ± 0.1	p<0.05
Sodium/potassium ratio (mEq/mEq)				
n	101	101	101	
Crude	4.0 ± 0.2	4.2 ± 0.2	4.1 ± 0.2	NS
Age and gender adjusted	3.9 ± 0.2	4.2 ± 0.2	4.2 ± 0.2	NS

Abbreviations: n, number; 24U, 24-hour urine; 24UT, 24-hour urinary taurine; T, taurine.

^1^ From multiple linear regression models for the relationship between 24UI/creatinine ratio and cardiovascular risks, fasting blood and 24U.

^2^Mean ± standard error (SE) (all such values).

After the adjustment for age and sex, significant differences were noted in four items in T3, which showed significantly higher TC, HDL-C, 24UNa and 24UK (S1 Fig).

#### Soy intake and cardiometabolic risks

A total of 303 participants, aged 30 to 79 years, were divided into tertiles I1–I3. The data are presented in Table 2. Since 3 cases showed the same I/creatinine ratios at the highest and lowest range of I1 and I2, n became 100 and 102 in I1 and I2, respectively.

**Table 2:** Association of tertiles of biomarker of soy intake (24UI) with cardiovascular risks, fasting blood, and 24U in Japanese, Hyogo inhabitants aged 30 to 79 Years.

	Tertile of 24UI/Creatinine ratio, mmol/mmol			P for linear trend^1^
	1 (low)	2	3 (high)	
Median	0.3	1.1	3.6	
Range	0.0–0.6	0.6–1.9	2.0–15.6	
Body mass Index				
n	100	102	101	
Crude	22.4 ± 0.3^2^	22.8 ± 0.3	22.2 ± 0.3	NS
Age and gender adjusted	22.4 ± 0.3	22.8 ± 0.3	22.2 ± 0.3	NS
Systolic blood pressure (mmHg)				
n	100	102	101	
Crude	121.9 ± 1.6	122.1 ± 1.8	123.9 ± 1.6	NS
Age and gender adjusted	123.2 ± 1.6	122.6 ± 1.5	121.9 ± 1.6	NS
Diastolic blood pressure (mmHg)				
n	100	102	101	
Crude	73.5 ± 1.2	73.9 ± 1.2	72.4 ± 1.1	NS
Age and gender adjusted	73.1 ± 1.2	74.1 ± 1.1	72.6 ± 1.2	NS
Serum total cholesterol (mg/dL)				
n	100	102	101	
Crude	202.7 ± 3.4	201.5 ± 3.4	216.0 ± 3.6	p<0.01
Age and gender adjusted	208.5 ± 3.4	202.5 ± 3.3	209.2 ± 3.4	NS
Low-density lipoprotein cholesterol (mg/dL)				
n	100	102	101	
Crude	120.6 ± 3.2	119.6 ± 3.1	129.3 ± 3.3	p<0.05
Age and gender adjusted	125.7 ± 3.1	120.6 ± 3.0	123.2 ± 3.1	NS
High-density lipoprotein cholesterol (mg/dL)				
n	100	102	101	
Crude	61.9 ± 1.4	62.2 ± 1.6	65.0 ± 1.4	NS
Age and gender adjusted	62.4 ± 1.5	62.0 ± 1.4	64.8 ± 1.5	NS
Triglyceride (mg/dL)				
n	95	100	97	
Crude	104.2 ± 6.8	105.2 ± 7.3	108.3 ± 7.4	NS
Age and gender adjusted	102.9 ± 7.3	106.0 ± 6.9	108.7 ± 7.4	NS
Folate (ng/mL)				
n	100	102	101	
Crude	5.8 ± 0.3	6.4 ± 0.3	8.4 ± 0.4	p<0.01
Age and gender adjusted	6.0 ± 0.3	6.4 ± 0.3	8.1 ± 0.3	p<0.01
24U Salt (g/day)				
n	100	102	101	
Crude	10.5 ± 0.4	11.5 ± 0.4	11.6 ± 0.5	NS
Age and gender adjusted	9.9 ± 0.5	11.4 ± 0.4	12.3 ± 0.5	p<0.001
24U potassium (g/day)				
n	100	102	101	
Crude	1.7 ± 0.1	1.9 ± 0.1	2.4 ± 0.1	p<0.001
Age and gender adjusted	1.7 ± 0.1	1.9 ± 0.1	2.3 ± 0.1	p<0.001
Sodium/potassium ratio (mEq/mEq)				
n	100	102	101	
Crude	4.5 ± 0.2	4.3 ± 0.2	3.5 ± 0.2	p<0.001
Age and gender adjusted	4.2 ± 0.2	4.2 ± 0.2	3.8 ± 0.2	NS

Abbreviations: n, number; 24U, 24-hour urine; 24UI, 24-hour urinary isoflavones.

^1^ From multiple linear regression models for the relationship between 24UI/creatinine ratio and cardiovascular risks, fasting blood, and 24U.

^2^Mean ± standard error (SE) (all such values).

After the adjustment for age and sex, significant differences were noted in only 3 items in I3, which showed significantly higher folate, 24UNa and 24UK than I1 (S2 Fig).

#### Concomitant fish and soy intakes and cardiovascular risks

A total of 303 were divided into 9 groups, T1–3 x I1–3. Among the 9, T1I1, T2I2, and T3I3 are compared in Table 3.

**Table 3:** Association of tertiles of biomarkers of fish and soy intakes (24UT, 24UI) with cardiovascular risks, fasting blood, and 24U in Japanese, Hyogo inhabitants aged 30 to 79 Years.

	Combination of tertiles of 24UI/Creatinine ratio and 24UT/Creatinine ratio			P for linear trend^1^
	T1I1 (low)	T2I2	T3I3 (high)	
Body mass Index				
n	37	31	43	
Crude	22.2 ± 0.5^2^	22.9 ± 0.5	21.6 ± 0.6	NS
Age and gender adjusted	21.8 ± 0.6	22.9 ± 0.6	21.9 ± 0.5	NS
Systolic blood pressure (mmHg)				
n	37	31	43	
Crude	119.9 ± 2.8	124.7 ± 3.0	122.6 ± 2.4	NS
Age and gender adjusted	121.9 ± 2.6	123.7 ± 2.7	121.5 ± 2.4	NS
Diastolic blood pressure (mmHg)				
n	37	31	43	
Crude	70.8 ± 2.0	74.0 ± 2.0	71.9 ± 1.7	NS
Age and gender adjusted	71.0 ± 1.8	73.6 ± 1.9	72.1 ± 1.6	NS
Serum total cholesterol (mg/dL)				
n	37	31	43	
Crude	194.4 ± 5.3	203.5 ± 6.9	219.4 ± 6.3	p<0.01
Age and gender adjusted	204.5 ± 6.3	200.9 ± 6.4	212.5 ± 5.6	NS
Low-density lipoprotein cholesterol (mg/dL)				
n	37	31	43	
Crude	116.8 ± 4.7	123.4 ± 6.7	132.4 ± 5.4	p<0.05
Age and gender adjusted	126.7 ± 5.5	120.7 ± 5.5	125.8 ± 4.9	NS
High-density lipoprotein cholesterol (mg/dL)				
n	37	31	43	
Crude	61.4 ± 2.1	63.1 ± 2.9	67.5 ± 2.3	NS
Age and gender adjusted	60.1 ± 2.5	63.7 ± 2.6	68.1 ± 2.3	p<0.05
Triglyceride (mg/dL)				
n	34	31	43	
Crude	91.0 ± 10.4	99.9 ± 8.0	92.5 ± 6.3	NS
Age and gender adjusted	95.1 ± 8.7	97.8 ± 8.5	90.8 ± 7.5	NS
Folate (ng/mL)				
n	37	31	43	
Crude	5.8 ± 0.5	6.9 ± 0.5	9.5 ± 0.7	p<0.001
Age and gender adjusted	6.1 ± 0.7	6.8 ± 0.7	9.2 ± 0.6	p<0.001
24U Salt (g/day)				
n	37	31	43	
Crude	9.1 ± 0.6	12.7 ± 0.9	11.9 ± 0.7	p<0.01
Age and gender adjusted	8.1 ± 0.8	12.9 ± 0.8	12.6 ± 0.7	p<0.001
24U potassium (g/day)				
n	37	31	43	
Crude	1.5 ± 0.1	2.0 ± 0.1	2.5 ± 0.1	p<0.001
Age and gender adjusted	1.6 ± 0.1	2.0 ± 0.1	2.4 ± 0.1	p<0.001
Sodium/potassium ratio (mEq/mEq)				
n	37	31	43	
Crude	4.3 ± 0.4	4.4 ± 0.3	3.4 ± 0.2	p<0.05
Age and gender adjusted	3.8 ± 0.3	4.5 ± 0.3	3.8 ± 0.3	NS

Abbreviations: n, number; 24U, 24-hour urine; 24UT, 24-hour urinary taurine; 24UI, 24-hour urinary isoflavones; T, taurine; I, isoflavones.

^1^From multiple linear regression models for the relationship between 24UT and 24UI and cardiovascular disease risks, fasting blood and 24U.

^2^Mean ± standard error (all such values).

**Fig 2:**
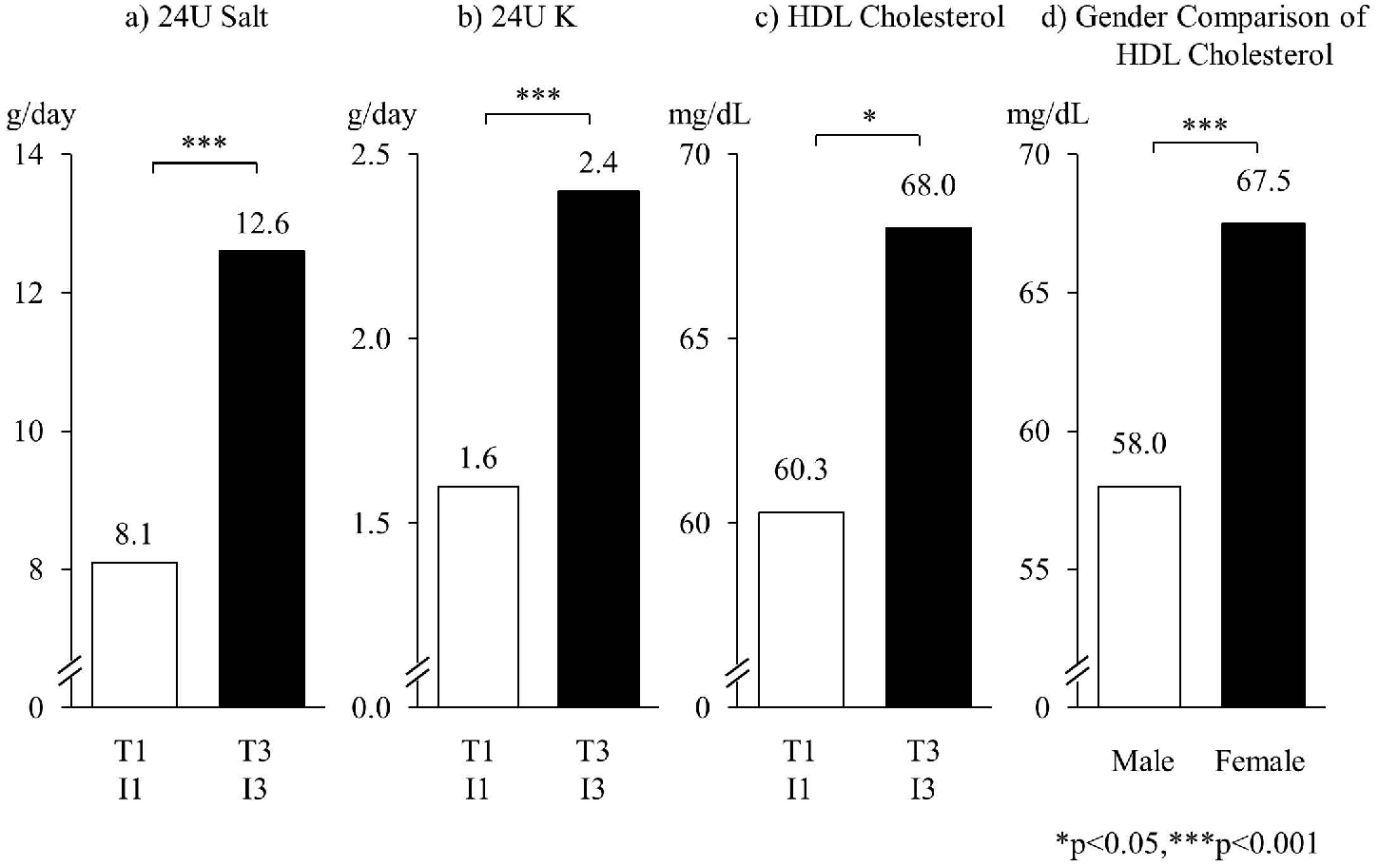
Concommitant fish and soy intake related to 24U salt and potassium excretions and to HDL cholesterol, in comparison with difference in HDL by gender. Abbreviations: 24U, 24hour urine; K,potassium; HDL,high-density lipoprotein; T1(3),lowest(highest) tertile of 24U taurine/creatinine;I1(3), lowest(highest) tertile of 24U isoflavone/creatinine. a) and b): adjusted for age and gender, c):adjusted for age, gender and hypolipidemic agents, d) adjusted for age.

**Fig 3:**
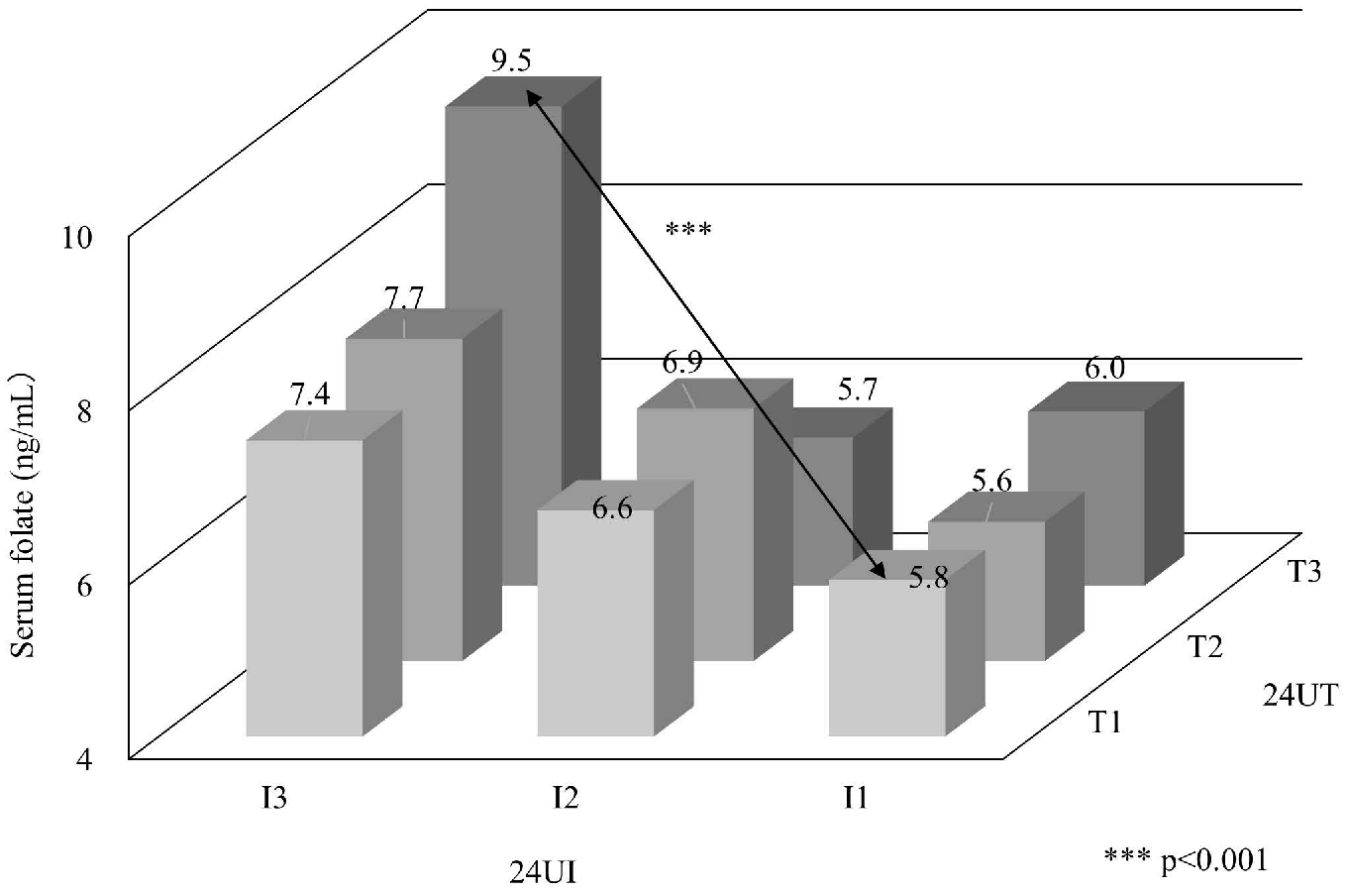
Fish and soy intakes related to serum folate in Japanese, Hyogo inhabitants aged 30–79. Abbreviations: 24UT, 24hour urinary taurine; 24UI, 24hour urinary isoflavone; T1-3, lowest to highest tertile of 24U taurine/creatinine;I1-3,lowest to highest tertile of 24U isoflavone/creatinine.

After the adjustment for age and gender, the concomitantly high T and I group, T3I3 was significantly higher than the concomitantly low T1I1 group in 4 items, HDL-C, folate, 24UNa, and 24UK. High fish and soy consumers were taking significantly higher salt intake, 12.6 g/day compared with low consumers, 8.1 g/day. Despite the high Na intake, the Na/K ratio was not significantly high due to the significantly high 24UK. The difference between T3I3 and T1I1 in HDL-C was close to the gender difference in HDL-C of the studied population (Fig 2). Folate level in T3I3 was highest among 9 groups, T1–3 × I1–3 (Fig 3). These findings are related to lower cardiovascular risks with fish and soy intakes (Figs 2 and 3).

## Discussion

The CARDIAC study demonstrated that two biomarkers of fish intakes (① 24UT and ② n-3 polyunsaturated fatty acid ratios of total fatty acids in the plasma phospholipids) were inversely related to CHD mortality rates in worldwide populations [9–11]. In addition, the biomarker of soy intake, 24UI, was inversely related to CHD mortality rates [10,11]. Since CHD mortality rates were inversely related to the average life expectancy in the world [11], Japan’s position at the top in life expectancy appears to be due to having the lowest CHD mortality rates among the developed countries. Therefore, the Japanese custom of consuming fish and soy, which reduces CHD mortality rates, would contribute to longevity in Japan.

The present study revealed that the Japanese were customarily consuming fish and soy. However, if, the lower CHD mortality rates were related to ethnic characteristics of the Japanese, either genetic or environmental, the greater intake of fish and soy would not necessarily be related to the lower CHD mortality in the Japanese population. Therefore, in this study, CHD risks were studied among the Japanese themselves, among whom there were differences in the intake of fish and soy.

The beneficial effects of fish nutrients such as n-3 polyunsaturated fatty acids and T were clearly demonstrated also by the inverse association of these markers with CHD mortality rates [9–11]; and the reduction of CHD risks due to, for example, blood pressure and lipid profiles is also well-documented [14–19]. The blood pressure-lowering effect of fish intake may be attenuated in this study by the concomitant high salt intake of fish-eating Japanese, as proved by the significant positive association between 24UT and 24UNa. The elevated serum total cholesterol in the third tertile, T3, compared with T1, noted after the adjustment for age and sex, was also reported by another intervention study after fish oil intake, and the mechanisms had been described [20,21]. The present study indicated that in those with a high consumption of fish (T3), LDL cholesterol was not significantly higher than in those with a low consumption of fish (T1), either in crude or in age-gender adjusted analysis. HDL-C was proved to be significantly increased in T3, confirming the merit of fish intake against CHD. The antiatherosclerotic effect of HDL-C has been well documented [22] and the mechanisms have recently been discussed anew for atherosclerosis treatment by increasing HDL-C [23].

The CARDIAC study showed inverse associations of 24UI with CHD mortality rates as well as with mortality rates due to prostate cancers in men and breast cancer in women [10,11,14]. In the present study, limited only to the Japanese, the tertiles of 24UI did not show any significant differences directly related to CHD risks such as blood pressure and cholesterol levels. This may be due to the relatively high average 24UI level in the Japanese compared to populations taking no soy diets, as shown in the CARDIAC study. The other reasons for finding no significant differences in blood pressure and lipid profile among the 3 groups of different soy intakes may be due to individual differences in the metabolism of isoflavones such as equol producers or non-producers [24] or in the genotype of Apolipoprotein E [25]. However, the highest soy consumption group, I3, showed significantly higher blood folate levels in crude data as well as after age and sex adjustment. Soy foods contain folate, and therefore, folate levels are increased in those who consume soy, whose homocysteine levels were proved to be low [26]. The intervention studies on the effect of soy protein diets reported the reduction of homocysteine and other CHD risks in type 2 diabetes [27] and hyperlipidemic subjects [28]. Soy protein in low in sulfur aminoacids and would result in lower homocysteine levels. Since homocysteine is atherogenic [29], soy intakes are regarded as being antiatherogenic.

The T3I3 group, Japanese who customarily consumed both fish and soy products, was proved to have high HDL-C and folate levels after the adjustment for age, sex, and hypolipidemic agents. The difference in HDL-C (7.7 mg/dL) between T3I3 (68.0) and T1I1 (60.3) was close to the sex difference (9.5), high in women and low in men, as was also observed in this study (Fig 2). Therefore, the cardioprotective effect of the increased HDL-C due to an ample intake of fish and soy may contribute to an increase in life expectancy, which is 7 years longer in Japanese females than in males.

The T3I3 group showed also an obviously higher folate level (9.5 ng/mL) than T1I1 (5.8). High folate levels decrease homocysteine and oxidative stress, and therefore, decreases atherosclerotic lesions [30]. Extensive epidemiological studies in Japan indicated an inverse association of folate intake with CHD diseases [31]. After the folate fortification of grains was fully implemented in the United States and Canada in 1998, stroke mortality was proved to be reduced [32,33].

Moreover, the T3I3 group was significantly higher in HDL-C than T1I1. The anti-atherosclerotic effect of HDL-C also corroborates with the effects of folate. Some intervention studies have shown that the effects of soy products or fish oil, and their combined effects on reducing cardio-metabolic risks, were related to lipid profiles and glucose metabolism [21,34].

However, Japanese individuals who consume soy products and fish are accustomed to a high intake of Na. The adverse effect of Na is well documented, including in the CARDIAC study, which clearly indicated a significant positive association of 24UNa with stroke mortality [9–11].

K intakes were high in those who consume soy and fish, but the Na/K ratio, which was related significantly with stroke mortality rates in the CARDIAC study [11], was not significantly high in the population consuming soy and fish, due to the high K intake of that population. Therefore, the Japanese custom of taking both soy and fish is beneficially anti-atherosclerotic because of high HDL-C and folate levels, and reduces the mortality from CHD, thus to contribute to the extension of average life expectancy.

However, the accompanying high Na intake of soy and fish diets can cause high blood pressure and increase the risk of strokes, causing premature disability and therefore, shortens healthy life expectancy. In conclusion, low-salt soy products and fish are recommended for healthy longevity in Japan as well as in the rest of the world, where the mortality rate of non-communicable diseases is more than 60% [35].

The strength of this study was first the objective estimation of the intake of fish and soy using 24U analysis of their respective biomarkers, T and I, and this was the first finding that nutritionally characterized Japanese diet in comparison with a worldwide population of samples. Second, the Japanese study population was selected from cities, coastal, and mountain areas of Hyogo Prefecture, which is located in the middle of Japan and often regarded as “a Japan in miniature” [36] because of the climate and 3 different geographical zones (urban, seaside, and mountainous) in the prefecture.

The limitation of this study was first the number of 24U samples that was analyzed for all items was limited. Since T was not measured in some samples, only the samples with complete data including both T and I were used for the analysis. Second, the years of study in the WHO study and Japanese studies in Hyogo Prefecture were different. The CARDIAC study’s health surveys, which started in 1985 and continued over 10 years in 50 populations, were utilized to characterize the Japanese diet by 24U biomarkers. The Japanese populations in Hyogo Prefecture were studied in 2011 and 2012. Since Japanese women’s average life expectancy became the top in the world in 1985, figures for both Japanese men and women marked all-time highs up to the present [1,2]. Therefore, despite this difference in study years, we may conclude that thse characteristic dietary factors of Japanese individuals have been proven to reduce the risk of CHD and thus, contributed to the longevity of the Japanese population, which has been maintained at the top in the world for those 30 years.

The conclusion of this study recommends more soy and fish consumption for health promotion throughout the world. Soy beans and other protein-containing grains are regarded as an eco-friendly source of protein sustainably available and able to support about 10 times the number of people who customarily get protein mainly from beef and pork. [37] The recommendation for fish consumption throughout the world, however, may lead to an exhaustion of fish stocks from our planet. This environmental concern may hopefully be solved by seaweed consumption, which is as high as 5.3g per day in Japan [38]. Some seaweeds traditionally eaten in Japan contain taurine as well as n-3 polyunsaturated fatty acids [39]. Our previous intervention study on providing 3g of taurine a day to Tibetan vegetarians decreased the prevalence of hypertension in 2 months [40]. Seaweed abundantly available in the sea may therefore be recommended for vegetarians so as to get these beneficial nutrients to them. For the sustainable development of eco-friendly global health, therefore, soybeans and seaweed, in addition to fish, may be recommended to achieve healthy longevity through cardiovascular disease prevention throughout the world.

## Supporting information

S1 Fig Tertiles of taurine (Tau)/Cre and HDL-cholesterol, 24U K and salt. (DOCX)

S2 Fig Tertiles of isoflavone (Iso) /Cre and serum folate, 24hU pottasium and salt. (DOCX)
