# Correction: Soy and fish as features of the Japanese diet and cardiovascular disease risks

**DOI:** 10.1371/journal.pone.0186533

**Published:** 2017-10-11

**Authors:** Yukio Yamori, Miki Sagara, Yoshimi Arai, Hitomi Kobayashi, Kazumi Kishimoto, Ikuko Matsuno, Hideki Mori, Mari Mori

The figure legend for Fig 1 is incorrect. I5 in the figure legend is missing. The figure caption is also missing. Please see the complete and correct Fig 1 here.

**Fig 1:**
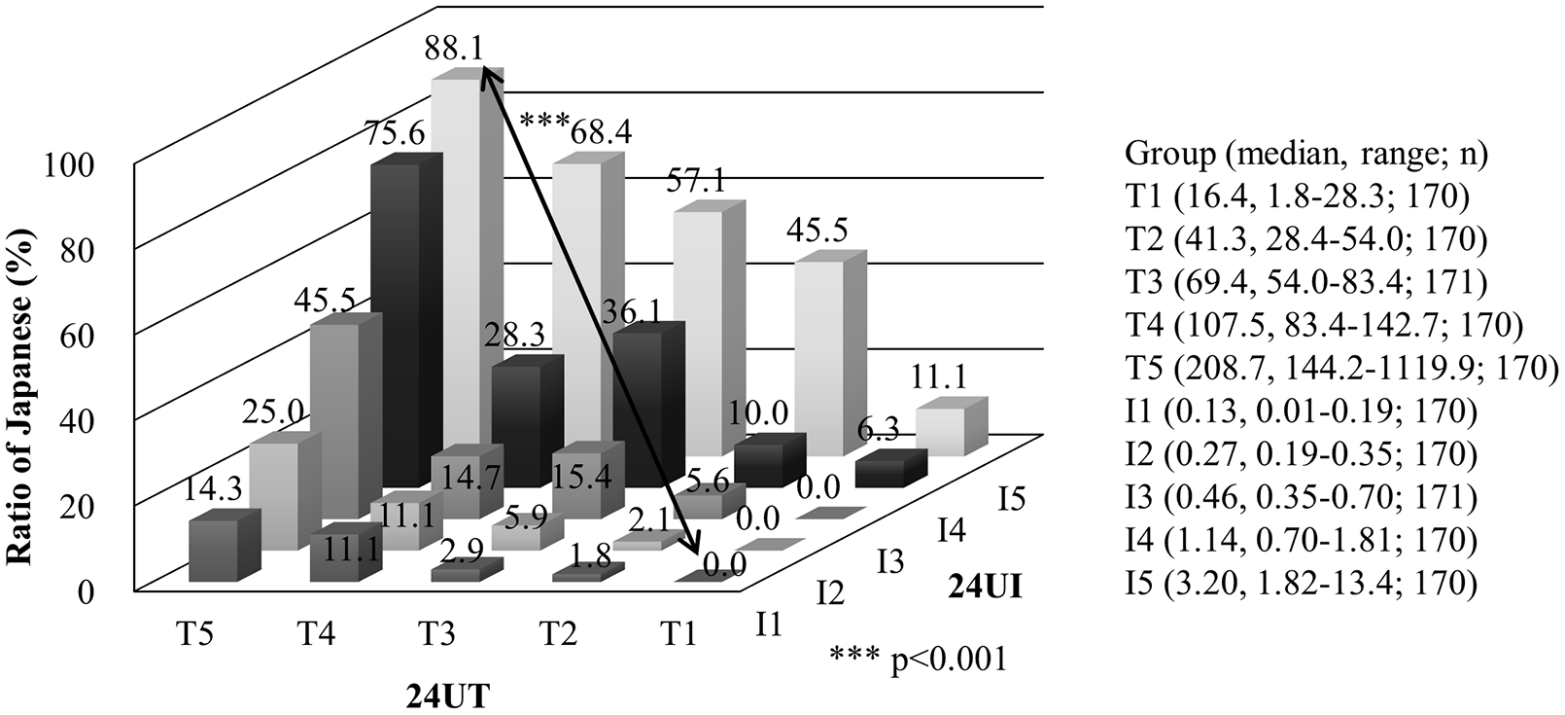
Ratio of Japanese in each quintile of biomarkers of Fish and Soy intake; 24 hour urinary (24U) taurine/creatinine (T) and isoflavone/creatinine (I) ratios. Abbreviations: 24UT, 24 hour urinary taurine; 24UI, 24 hour urinary isoflavone; T1-5, lowest to highest quintile of 24U taurine/creatinine; I1-5, lowest to highest quintile of 24U isoflavone/creatinine.

Additonally, the Supporting Information files S1 and S2 Figs have tracked changes. The final versions of S1 and S2 Figs are included below.

## Supporting information

S1 Fig Tertiles of Taurine (Tau)/Cre and HDL-cholesterol, 24U Potassium and Salt. (DOCX)

S2 Fig Tertiles of Isoflavone (Iso) /Cre and Serum Folate, 24U Potassium and Salt. (DOCX)

## References

[1] Yamori Y, Sagara M, Arai Y, Kobayashi H, Kishimoto K, Matsuno I, et al (2017) Soy and fish as features of the Japanese diet and cardiovascular disease risks. PLoS ONE 12(4): e0176039 https://doi.org/10.1371/journal.pone.0176039 28430815 10.1371/journal.pone.0176039 PMC5400241

