# Supplementary material for: Soy and fish as features of the Japanese diet and cardiovascular disease risks
Source: PLoS One. 2017 Apr 21;12(4):e0176039. doi: 10.1371/journal.pone.0176039 (PMC5400241; doi:10.1371/journal.pone.0176039)
Supplement: S1 Fig — (DOCX) [file pone.0176039.s001.docx]

**Supporting　information**

**S1 Fig. This is the S1 Fig Title. Tertiles of Taurine (Tau)/Cre and HDL-cholesterol, 24U K and Salt**
