# Supplementary material for: Soy and fish as features of the Japanese diet and cardiovascular disease risks
Source: PLoS One. 2017 Apr 21;12(4):e0176039. doi: 10.1371/journal.pone.0176039 (PMC5400241; doi:10.1371/journal.pone.0176039)
Supplement: S2 Fig — (DOCX) [file pone.0176039.s002.docx]

**Supporting　information**

**S2 Fig. This is the S2 Fig Title. Tertiles of Isoflavone (Iso) /Cre and Serum Folate, 24hU Pottasium and Salt**
