# Supplementary material for: Correction: Soy and fish as features of the Japanese diet and cardiovascular disease risks
Source: PLoS One. 2017 Oct 11;12(10):e0186533. doi: 10.1371/journal.pone.0186533 (PMC5636157; doi:10.1371/journal.pone.0186533)

**Supporting information**

**S1 Fig. Tertiles of Taurine (Tau)/Cre and HDL-cholesterol, 24U Potassium and Salt**


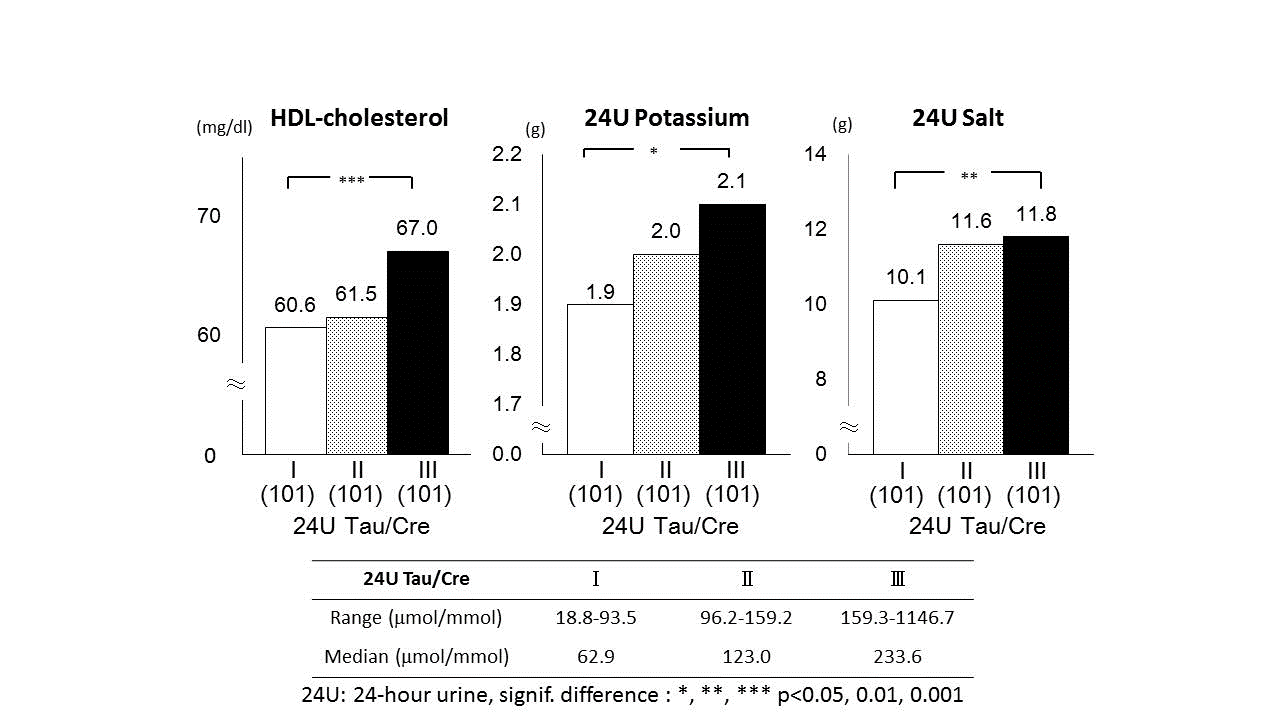

Supplement: S1 Fig — (DOCX) [file pone.0186533.s001.docx]
